# Plant-Based Meat Alternatives: Technological, Nutritional, Environmental, Market, and Social Challenges and Opportunities

**DOI:** 10.3390/nu15020452

**Published:** 2023-01-15

**Authors:** Giulia Andreani, Giovanni Sogari, Alessandra Marti, Federico Froldi, Hans Dagevos, Daniela Martini

**Affiliations:** 1 Department of Food and Drug, University of Parma, 43124 Parma, Italy; 2 Department of Food, Environmental and Nutritional Sciences (DeFENS), Università degli Studi di Milano, 20133 Milan, Italy; 3 Department of Animal Science, Food and Nutrition (DiANA), Università Cattolica del Sacro Cuore, 29122 Piacenza, Italy; 4 Wageningen Economic Research, Wageningen University and Research, 2595 BM The Hague, The Netherlands

**Keywords:** alternative proteins, consumer acceptance, flexitarianism, meat analogs, sustainability, SDGs

## Abstract

There is a growing awareness that fostering the transition toward plant-based diets with reduced meat consumption levels is essential to alleviating the detrimental impacts of the food system on the planet and to improving human health and animal welfare. The reduction in average meat intake may be reached via many possible ways, one possibility being the increased consumption of plant-based meat alternatives (PBMAs). For this reason, in recent years, hundreds of products have been launched on the market with sensory attributes (i.e., taste, texture, appearance, and smell) similar to their animal counterparts; however, these products have often a long list of ingredients and their nutritional values are very different from animal meat. The present review aims to highlight the main opportunities and challenges related to the production and consumption of PBMAs through an interdisciplinary approach. Aspects related to the production technology, nutritional profiles, potential impacts on health and the environment, and the current market and consumer acceptance of PBMAs are discussed. Focusing on the growing literature on this topic, this review will also highlight research gaps related to PBMAs that should be considered in the future, possibly through the collaboration of different stakeholders that can support the transition toward sustainable plant-based diets.

## 1. Introduction

It is broadly agreed that transitioning away from meat-intensive diets toward increasingly plant-based diets is essential to alleviating the adverse environmental sustainability impacts of the food system and to improving human health and animal welfare. However, our collective meat consumption is still increasing and is projected to keep rising in the coming decade [1]. To curb the projected global rise in meat consumption, it is argued that a substantial reduction in average meat consumption levels—starting in affluent societies—is critically important.

Meat reduction can be established in various ways: (i) reducing meat portion sizes, (ii) replacing parts of meat-based products with plant-based alternatives (so-called hybrid meats) or applying a “less but better” principle (less quantity, more quality, i.e., more environmentally and/or animal-friendly meat), (iii) leaving the meat out of the dish without a replacement, (iv) replacing meat with another protein source (ranging from animal-based foods, such as eggs or cheese, to plant-derived alternatives, such as legumes, mushrooms, or tofu—not to mention alternative sources of protein with a minimal, i.e., insects and seaweed, or still non-existent market share, i.e., cultured meat), and last but not least, (v) consuming plant-based meat alternatives (PBMAs) [2,3]. These strategies imply that a flexitarian diet should not be narrowed down to the adoption of (processed) meat alternatives because it is also about substituting meat with other (unprocessed) alternative proteins, both animal- and plant-sourced. Having said this, the broad and varied dietary group of flexitarians is undeniably the key target group of PBMAs and the major group already consuming these products. From the perspective of a flexitarian diet characterized by abstaining from meat (whether this is occasionally, frequently, or often), it is obvious that flexitarians are searching for and interested in meat alternatives to practice their reduced meat foodstyle. Briefly put, there is logic in pointing to flexitarians as launching customers. From the perspective of PBMAs, the dominant market strategy hitherto is to mimic traditional meat as closely as possible in terms of flavor (meaty/savory), texture (mouthfeel), appearance (e.g., “the bleeding burger”), nutritional value (iron, vitamins, etc.), and even product names (using meat-related terms); incidentally, “PBMAs” may also be read as “plant-based meat analogs”. The food industry’s goal to develop meat-like plant-based foods unquestionably facilitates the meat-free choices of many flexitarians and vegetarians and vegans as well, who may feel an aversion to the association with meat surrounding PBMAs.

While food consumers’ adoption and acceptance of PBMAs are not self-evident, as will be shown in the remainder of this review, it seems safe to say that PBMAs facilitate the need of many of today’s food consumers in various high-income countries to be supplied with tasty, affordable, and accessible alternative protein products to satisfy their cravings to eat beyond meat.

Currently, many factors can testify that the field of PBMAs is vibrant and worth being further explored and critically assessed. Among these factors are the remarkable successes of efforts to improve the product qualities of PBMAs in the past few decades and the wide availability of PBMAs on supermarket shelves and in the food service sector (including McDonald’s, Burger King, and KFC, having released plant-based alternative versions of beef burgers and chicken nuggets). Furthermore, the substantial investments in the PBMA market, the significant growth figures it is experiencing in frontrunning countries (such as Germany, the UK, and the Netherlands), and its expected growth rates in global sales in the near future are additional elements that can attest to the key role of PBMAs.

This review aims at highlighting the main challenges and opportunities related to the production and consumption of PBMA products, taking into consideration all of the pivotal aspects of designing new food products. Indeed, after a brief excursus on the formulation and production technology of PBMAs (i), the review addresses their nutritional profiles and their potential impacts on health (ii) and the environment (iii), as well as consumers’ choices (iv) and the state of the market (v). Each of the five sections will provide a sketch of the state of affairs, and overall, this article aims to add to other recent reviews [4,5] by critically assessing recent studies from different disciplines in order to highlight the consensus and controversies on this topic from an interdisciplinary perspective.

## 2. Production Technology of Plant-Based Meat Alternatives

Among the earliest examples of meat alternatives, vegetable protein products are traditionally produced and consumed in Asian countries—i.e., tofu and tempeh from soy and seitan from gluten. Unfortunately, these products are not able to replicate the sensory attributes of meat products for Western consumers, who seek vegetable-based products that resemble meat in structure, flavor, and taste. Twenty-first-century meat alternatives have made use of the crosslinking capacity—under certain conditions—of soy proteins from the Asian tradition. Indeed, even today, soy is the main raw material for the production of meat alternatives [6]. This supremacy undoubtedly depends on the availability of the raw material and the techno-functional attributes of its proteins, including its solubility, its ability to absorb water and oil, and its gelling and emulsifying properties—all important aspects in defining the quality of the finished product [7]. However, scientific research (and the market) is shifting toward the use of raw materials other than soy because of issues concerning GMOs, allergies, unfavorable climate for soy cultivation, and the preservation and/or valorization of biodiversity. Thus, recent work explored the use of proteins from different raw materials, including peas, fava beans, rapeseeds, and hemp, alone or in combination with soybean [8]. Regardless of the botanical source, protein isolates—with protein content above 75% (usually close to 90%)—are the most used raw materials [9].

Protein isolates are produced using wet separation techniques that are often time-consuming, costly, inefficient, and unsustainable, given the high amounts of water, alkalis, acids, or enzymes employed [9]. Finally, since the functionality of proteins can widely vary depending on the process conditions adopted during protein isolation, the standardization of the technological properties of the isolates is challenging [8]. Thus, protein isolates are increasingly being replaced by protein concentrates (protein content between approx. 50 and 65%), without neglecting the structural properties required in the finished product [10]. These high-protein fractions are produced using dry separation processes. The latter type of process is considered more sustainable than wet techniques, as it requires no water or solvents, consumes less energy, and preserves the protein’s native structure instead of forming protein aggregates, thus retaining their technological functionality [8]. The principle behind the air classification is the different densities of the flour particles, which are richer in starch or proteins. This allows the separation of the flour into a fine protein-rich fraction and a coarse starch-rich fraction as a consequence of the centrifugal and gravitational forces applied during the operation. Therefore, the less-refined protein ingredients obtained with the air classification also contain other components, such as lipids and fibers, which are often included in the formulation of protein-isolate-based products [9]. Since the lipid and fiber contents in protein concentrates may vary based on the source and processing conditions, the set-up of the air-classification conditions needs to be optimized. So far, there are few examples—albeit with encouraging results—of the application of high-protein fractions obtained using air separation from legumes in the production of meat analogs [11], suggesting the need for further studies that also use different sources.

In order to expand the range of raw materials that are suitable to be used in the production of meat alternatives and that can maintain the high-quality characteristics of the finished product, various colorants (e.g., leghemoglobin, red beets, red cabbage, etc.) and flavorings (e.g., herbs and spices) have been proposed to reproduce the meat color and flavor profile, as well as to mask the beany off-flavors of some legume proteins. The juiciness, tenderness, and other sensory attributes of meat-like products are also obtained by using fats/oils (such as coconut oil/butter, sunflower oil, canola oil, sesame oil, etc.). However, it is increasingly common to use binding agents (e.g., oleogels, starches, hydrocolloids, or fibers) as fat replacers [10]. Indeed, high amounts of fat—acting as a lubricant—could interfere with the protein denaturation process, which is the first kind of modification proteins need to undergo in order to obtain a meat-like structure.

The meat-like structure is achieved when the native globular structure of pulse proteins is transformed into a fibrous structure in which proteins are elongated and highly ordered [8]. This structure can be created using different technologies (including extrusion, flow-induced structuring using a shear cell or a Couette cell, 3D printing, wet-spinning, and electrospinning), the advantages and disadvantages of which were recently summarized by Boukid [12].

The high productivity, low costs, versatility, energy efficiency, and scale-up potentials of extrusion have led it to be the most widely used technology to produce meat analogs. During this process, raw materials are hydrated and subjected to thermal and mechanical stresses applied during extrusion, and, finally, the product is cooled to room temperature [13]. As a result of the mechanical stress, the temperature, the pressure, and the final cooling step, proteins undergo a series of structural modifications, ranging from denaturation to unfolding, crosslinking, and alignment, resulting in a fibrous structure that mimics the characteristics of muscle tissues [14]. These modifications take place in a chamber containing one (i.e., single-screw extruder) or—more commonly—two (i.e., twin-screw extruder) corotating screws that convey the material toward a die that provides the final shape to the product. The extrusion chamber is subdivided into several zones, in which the peculiar profiles of the screws—and, thus, the applied shear—and temperatures cause the material to undergo (from the material inlet to the finished product outlet) mixing, hydration, shearing, homogenization, compression, deaeration, heating, shaping, and expansion. During these operations, proteins are hydrated, unfolded, aligned, and texturized.

Extrusion can be performed at a low moisture level (<30%) to obtain texturized vegetable proteins (TVPs) or at a high moisture level (>50%) to directly obtain meat analogs. When extrusion is carried out at low moisture, the sudden drop in pressure at the end of the extruder causes an immediate expansion of the product due to the rapid evaporation of water. TVPs have a spongy meat-like structure that mimics ground beef or chicken breast. TVPs can take different forms (flakes, chunks, or minced), and, after hydration (and final cooking), they are able to retain their structural integrity and acquire a chewy texture and elasticity, which is typical of meat.

In the case of high-moisture extrusion, a cooling die is connected at the end of the twin-screw extruder to cool the sample at 20 °C, which prevents the expansion and promotion of fiber alignment and stabilization, as is typical of the anisotropic structure desirable for these kinds of products.

Although the use of technologies other than extrusion has shown encouraging results (including high-temperature-induced shearing and 3D printing), some hurdles still need to be addressed before their widespread industrial deployment: cost reduction and/or applicability to a wide range of legume proteins.

## 3. Nutritional Profiles and Health Impacts of Plant-Based Meat Alternatives

Among the several reasons related to the growing demand for meat alternatives, a potential explanation is likely related to the increased knowledge about the negative impacts of diets high in red meat and, above all, processed meat on human health [15]. This, together with an increased concern for the environmental impacts of animal products compared to their plant-based counterparts, supports the transition toward sustainable healthy diets, which are based on a high intake of plant-based foods and the moderate consumption of animal products [16].

However, to investigate the potential role of PBMAs on human health, it is critical to analyze the nutritional characteristics of these products, also considering that meat is an essential source of high-quality proteins, iron, vitamins, minerals, and varying amounts of saturated fats depending on the type of meat [17]. A few studies analyzed the nutritional quality of meat alternatives present in different markets and compared meat alternatives and animal meat in terms of energy and nutrient contents [18,19,20].

In this regard, a recent study analyzed the nutritional quality of 269 commercial meat analogs currently sold on the Italian market by retrieving data reported on their food labels [19]. Large nutritional variability was observed among PBMAs, with plant-based steaks showing significantly higher protein and lower energy, fats, and salt contents compared to other plant-based food categories. Comparing the nutritional information with reference animal meat products, the results showed higher fiber content in all PBMAs. Moreover, plant-based burgers and meatballs had a lower protein content than their meat counterparts, while ready-sliced meat substitutes showed a lower salt content than cured meats.

Similar results were obtained in other studies performed in the US [18], Sweden [20], and other European markets [12]. These studies found lower energy and total and saturated fat contents and higher total carbohydrates, sugars, and fibers in PBMAs compared to meat-based products. On the other hand, salt content showed contrasting results. Furthermore, plant-based and meat-based products generally presented similar amounts of total proteins despite large differences in the contents of single amino acids. As a matter of fact, higher amounts of glutamic acid and cysteine and lower contents of alanine, glycine, and, above all, methionine were identified in PBMAs [21].

These results support the importance of further exploring the use of plant-based protein blends to reduce differences between plant-based and animal-based meats [22]. In addition, it is noteworthy that plant-based and animal products also differ in protein digestibility and the bioavailability of single amino acids. Indeed, animal meat showed higher protein digestibility than PBMAs, which, in turn, have a negative impact on amino acid bioavailability. These data suggest the possibility to use specific protein sources with high bioavailability (e.g., soy isolate) and stress the importance of considering the real bioavailability of amino acids when investigating the diet quality of dietary patterns that include these products.

Another interesting aspect to be considered regards micronutrients. Data are often limited on this topic, but previous studies highlighted that PBMAs are a good source of minerals, also reporting a higher iron content compared to meat [21,23]. However, it is important to underline that the absorption and bioavailability of iron from plant-based sources and vegetarian diets are lower compared to omnivorous diets, and this shall be considered in future investigations [24].

Altogether, these results highlight the importance of carefully evaluating the nutritional impacts of switching from animal meat to PBMAs in order to identify potential at-risk nutrients. With this intention, a recent study compared the omnivore diet with diets in which animal products were substituted with either traditional or novel plant-based foods by using NHANES 2017–2018 data. The risk of inadequacies of specific nutrients (e.g., vitamin B12) was highlighted, especially when novel PBMAs were used [25]. These results once again support the need to consider the nutritional quality of PBMAs when switching to plant-based diets that exclude the consumption of animal foods.

Another area that deserves further investigation is the evaluation of the impact of replacing animal meat on human health through well-designed human intervention studies. So far, different studies have compared the effects of vegetarian/vegan diets with those of omnivorous diets [26], but trials specifically focused on PBMAs are still lacking. Yet, due to the publication of study protocols in clinical trial registries (e.g., ClinicalTrials.gov), it is reasonable to expect the implementation and publication of trials evaluating the impacts of PBMAs on nutritional and health aspects in the near future. A first attempt was recently made by Crimarco and colleagues [27], who assessed the effects of plant-based meats on biomarkers of inflammation through a secondary analysis of the Study With Appetizing Plant food—Meat Eating Alternatives Trial (SWAP-MEAT). Contrary to expectations, no improvements in biomarkers of inflammation following plant-based meat consumption were identified. However, further long-term studies focused on a large plethora of health markers are necessary before drawing any conclusions.

## 4. Environmental Impacts of Plant-Based Meat Alternatives

Meat is a protein food of high biological value; however, the conversion of feed and fodder into animal protein may not be sustainable due to inputs and the use of limited natural resources [28]. Currently, several farming systems of meat production exist, with the production efficiency per unit of a product depending mainly on feeding, breeds, management, and the technology employed [29,30]. Fewer resources per product unit are required for crop growth, which leads these products to represent an interesting opportunity for sustainable development while meeting the increasing demand for food [31]. Thus, in developed countries not relying on subsistence animal breeding, PBMAs could bring environmental benefits in terms of biodiversity, land and water use, and reduced greenhouse gas (GHG) emissions [32,33].

Nonetheless, the environmental impacts of PBMAs still need to be assessed. In this regard, the life cycle assessment (LCA) approach has been applied. It is a methodology used in various contexts to quantify the environmental impacts of a product based on the ISO 14040 [34] and ISO 14044 [35] standards to improve its environmental performance [36].

Several LCA studies were conducted on PBMAs to detect hotspots in the production process and to compare environmental performances with animal-based products. Indicators such as climate change, land, water, and energy use were considered.

In this regard, Bryant [37] analyzed 43 studies and concluded that the production of meat analogs is more sustainable when compared to animal products. At the same time, Detzel et al. [38] stated that PBMAs could help reduce the environmental impacts related to food consumption by overcoming the complexity of the processing stage of ingredients—which has a significant environmental impact—and by optimizing the inputs required to produce protein ingredients (i.e., legumes, trying to stabilize their yields, the main problem in their cultivation) [39]. Nevertheless, Smetana et al. [40] reported that the technology employed (i.e., machinery and process equipment) might be a valuable opportunity to improve the sustainability of alternative protein source production. A detailed LCA study by Mejia et al. [41] on three factories producing 57 different types of meat analogs achieved low GHG emissions, mainly due to the manufacturing process, followed by the agricultural production of food ingredients and their transportation. According to Goldstein et al. [42], the production stage accounts for 80% of the environmental impact due to the use of electricity from fossil sources; however, alternative energy solutions could mitigate this impact.

In-depth studies are needed since contrasting data are ascribed to energy consumption derived from the use of proxy processes for the implemented energy sources [37]. Within the meat supply chain, meat production and animal husbandry are the most impactful stages [43]. Nevertheless, manure production, subsequently applied to the soil, spares the need for chemical fertilizer, contributes to crop yield, and maintains soil fertility. On the other hand, legumes do not require nitrogen fertilization due to their ability to fix nitrogen from the atmosphere and at the root level [44]. This leads to lower N_2_O and NH_3_ emissions due to the non-use of manure and/or synthetic fertilizers.

Several studies have considered the impact of meat and meat analogs on the water used and the effects on eutrophication and acidification. In a study comparing patties with and without meat, Smetana et al. [45] estimated lower acidification and subsequent aquatic eutrophication for PBMAs. Similar conclusions were obtained by Heller et al. [46], who showed lower water use for plant-based patties. However, guidelines for water modeling are needed to avoid misleading interpretations based on erroneous comparisons.

Lusk et al. [47] produced a model to study both the economic and environmental effects of the use of alternative plant products over meat in the US. The reforestation of cropland and pastureland, as well as the conversion of land for crops grown for livestock feeding to crops for plant-based products, would result in the sequestration of 0.43 megatons of CO_2_ per year. The results imply an increase in crop yields to compensate for the reduction in available cropland. At the European level, Saget et al. [48] found a reduction in human–animal competition for land use for pea protein production and an 89% lower global warming potential. In more detail, in Germany, a 5% substitution of beef with pea proteins could lead to a 1% reduction in annual CO_2_ emissions. However, it is important to assert that agricultural activities impact 9.9% of global greenhouse gas emissions [49]. There could be scenarios of increased arable land to fulfill the growth of alternative meat products, even when deforestation is limited through environmental policies. The extensification of palm plantations in humid tropical countries could be an example, with an increased demand for coconut oil as an ingredient in plant-based beef substitutes [42].

It can be concluded that, still, few LCA studies have quantified the environmental impacts of meat alternatives, and many limitations related to the application of the methodology need to be addressed. Relevant considerations are that (i) PBMAs are highly processed foods, and thus, impacts associated with the use of different forms of energy counteract the low environmental impact associated with the production of plant-based ingredients [41]; (ii) the building of databases for the productive process of complex (multi-ingredient) foods should be a relevant point to focus on; (iii) a functional unit that does not consider the mass of a product but integrates primary nutrients should be implemented, along with a feature required when comparing LCA results from different studies/products [48,50]; (iv) the sustainability of PBMA production must take into consideration good agricultural practices, such as crop rotation, fertilizer, plant protection, and water use [38].

## 5. Consumer Behavior of Plant-Based Meat Alternatives

In the realm of meat alternatives, despite technological innovations and efforts to design processed plant-based products from different sources, one of the main challenges in successfully replacing animal prom ducts with plant-based ingredients is the re-creation of similar meat sensory properties. Moreover, communication about these new products and individual attributes (e.g., attitude and demographics) should be taken into consideration during the marketing stage—especially in those countries where meat and meat-based products have a key role in consumers’ minds, in terms of habits, culinary traditions, and culture [5,51]. Therefore, both sensory and consumer science can play an important role in understanding how consumers perceive PBMAs, including drivers of and barriers to their acceptance.

First, past studies showed that perceived sensory attributes and consumer acceptance are strongly influenced by the choice of plant/protein sources [52,53]. Therefore, what ingredients to use as a replacement for meat is an important factor to consider in the development of meat alternatives [54]. Early product developments mimicking processed meat products, for example, those from mycoproteins, have low sensory acceptance in terms of taste and texture [55]. This results in a low willingness to include such products as a real meat substitute for meat eaters [56]. As mentioned above, until a few years ago, the first generation of these products was mostly designed for vegetarians and vegans [55,57,58]. To achieve acceptability by a wider audience of meat eaters, the new generation of PBMAs shall be developed in a way that texture, appearance, aroma, and taste resemble those of equivalent authentic meat products, before, during, and after cooking [5,57]. Yet, reproducing the complex and delicate sensory profile of farmed meat can be challenging [14,59]. For instance, the color of plant-based products may diminish due to light or oxygen exposure, or the taste could be affected by lipid oxidation and cause undesirable characteristics [52]. Considering that the appearance of a product is generally the first element to be assessed, it is a critical determinant in food acceptance. Another challenge for these PBMAs is to recall the flavor of real meat while avoiding unpleasant flavors (e.g., bitter, burnt, and earthy) caused by the high level of legume protein [5]. Therefore, the need to mimic meat characteristics requires the use of many additives in the development stage [5]. As a result, the product packaging of PBMAs often includes a long list of unfamiliar ingredients [19], which could convey a sense of processed and unhealthy food among consumers. In particular, PBMAs that are high/ultra-processed could be associated with a certain unnaturalness of the product [60]. Thus, while reducing the gap between the sensory profiles of PBMAs and their meat equivalents might be important for some companies, the concept of product acceptance goes beyond merely sensory appreciation, including consumers’ perceptions. For example, low product familiarity with PBMAs—including the preparation/cooking method—is one of the most important product-related factors associated with consumer acceptance. This could potentially limit the expansion to the mainstream consumer market. Therefore, fully understanding consumers’ acceptance of PBMAs should require individuals to have a direct experience [4].

The most investigated meat category in consumer studies, including sensory tests, is burgers [53,61,62,63]. The reason is that traditional burgers are one of the most popular meat forms due to their composition (e.g., rich in proteins and fats), market availability, convenience, affordability, and sensory qualities [64,65]. Results consistently indicate that respondents generally prefer traditional meat products over their plant-based alternatives. For example, Grasso et al. [62] showed that individuals had higher sensory expectations for a beef burger than for a plant-based or hybrid patty; however, in terms of acceptability and purchase intentions, the hybrid one (60% beef and 40% vegetables) was the most preferred after the tasting.

In general, product familiarity is also often associated with higher acceptance. For instance, another study by Caputo et al. [53], which included a choice experiment with a blind–informed sensory study, showed that the beef burger, which had the highest degree of familiarity, also received the highest willingness to pay (WTP) compared to two PBMAs and one hybrid burger. They also found that, in the informed group, the preference and WTP for the plant-based patty labeled as “made with animal-like protein” exceeded those for the hybrid burger (70% beef and 30% mushrooms) and the plant-based burger “made with pea protein”. As reported by several studies, low prices of non-meat protein sources may act as a driver to accept such products [66]; however, it will probably take some years to reach price parity with traditional meat [4].

Regarding demographics, habits, and attitudinal factors, being pro-health, pro-sustainability, and young leads to higher acceptability toward PBMAs compared to other consumer segments [5]. For these reasons, health and environmental sustainability benefits could be included among the main drivers to try such products [66]. For instance, in a study by Sogari et al. [51], motivations to process both sustainability and nutrition information were a strong determinant driving the likelihood to purchase a hybrid meat–mushroom burger among US students. Other impacting factors could be the attitude toward meat analogs [67] and, more generally, consumer attitude toward food innovation [51]. On the other hand, the main personal-related barriers to acceptability are related to food and food technology neophobia [4,5], attachment to meat, and lower situational appropriateness of consuming non-meat protein sources [66].

Several studies have shown that heavy meat eaters might be less willing to substitute meat products for plant-based alternatives than flexitarians [68,69]. However, other studies suggested that the greater the number of consumers who are already familiar with plant-based products, the fewer the individuals who will seek products that are similar to meat from a sensory point of view [5]. This could be explained by the fact that vegetarians and vegans are not seeking meat sensory properties in plant-based products [70].

Finally, more knowledge about consumer acceptance of PBMAs is also helpful for legislators. For instance, in the EU, policymakers support the production and promotion of alternative meat substitutes and hybrid products by funding research programs toward more sustainable and alternative proteins, such as the Farm to Fork Strategy in the European Union [71]. Thus, understanding how consumers perceive such products is challenging for the food system, and developing meat alternatives with high consumer appeal requires the full integration of sensory and consumer research.

## 6. Market Analysis of Plant-Based Meat Alternatives

Given that the latest market trends of plant-based meat alternatives have not been deeply investigated, we conducted market research to identify the current direction of these products. Retailers and industries could benefit from the data retrieved from this analysis to design new products (in terms of ingredients, claims, labeling, etc.) to better shape their market strategies.

To analyze market trends of PBMAs, we used Mintel’s Global New Product Database (GNPD) [72], an online database for new products launched in selected countries. The same database was previously used in other research. For instance, several authors employed Mintel’s GNPD to investigate front-of-package information, food labeling schemes, ingredient profiles, and new launches of alternative meat products in the global market [65,73,74,75].

The objective of our analysis was to use the Mintel database to extract and explore different information on the latest market trends of PBMAs. In order to have an overview of recent years, we searched for new meat alternative launches over the past three years (from January 2019 to December 2021). The dataset was extracted on 26 October 2022, and the search strategy is described in Appendix A (Table A1).

The research returned 5155 results in the form of a spreadsheet, where each column reported different information, such as ingredients, claims, and nutritional values per 100 g. After cleaning the dataset to remove non-meat alternatives (e.g., fish or egg alternatives) using keywords (e.g., seafood, salmon, tuna, and egg) in the “Product” and “Description” columns, the final dataframe was analyzed using descriptive statistics.

**Figure 1:**
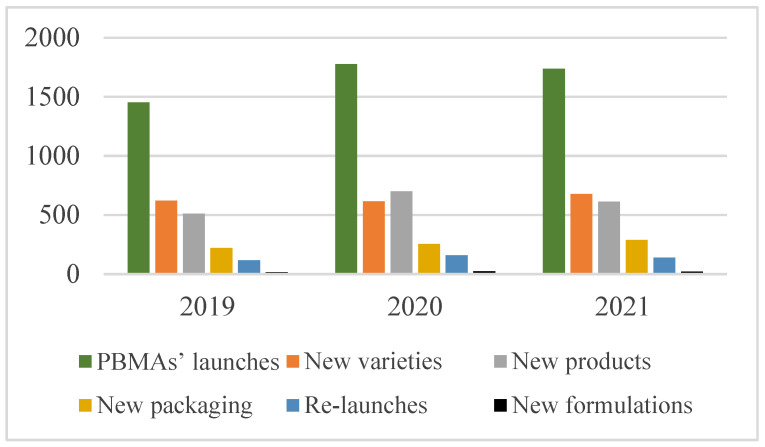
Number of PBMAs’ launches (*n* = 4965—green bar), new varieties (*n* = 1910—orange bar), new products (*n* = 1822—gray bar), new packaging (*n* = 1822—yellow bar), re-launches (*n* = 386—blue bar), and new formulations (*n* = 58—black bar) launched worldwide over the past three years (2019–2021). Abbreviations: PBMAs, plant-based meat alternatives.

During the past three years (2019–2021), the market of PBMAs has seen a remarkable spike in product launches, with 4965 products released worldwide. In more detail, Figure 1 shows the solid growth of PBMAs at the beginning of 2020—when the COVID-19 pandemic broke out—and a slight drop at the end of 2021. This change could be explained by common short-term reductions in meat intake during zoonotic outbreaks, as the same happened for SARS-CoV in 2003 and the African Swine Flu in 2019 (Attwood and Hajat, 2020). Thus, this meat intake reduction could have led consumers to look for new alternatives at the beginning of the coronavirus outbreak. Nevertheless, despite the modest negative trend during the third and fourth quarters of 2021, the overall direction of PBMA launches is positively growing, and this new dietary pattern could represent an opportunity to foster these products. More precisely, this positive market trend is mostly focused on the introduction of new products (*n* = 1822; 36.7%) and new varieties (*n* = 1910; 38.5%) of PBMAs. The remaining launches (*n* = 1232; 24.8%) include new packaging, re-launches, and new formulations.

It is also important to highlight that, despite the market for PBMA products experiencing increasing growth, the global market revenue of plant-based meat substitutes is forecast to be worth USD 33.99 billion in 2027 (Global: Meat Substitutes Market Revenue 2016–2027|Statista, 2022), while the meat sector is expected to be valued at USD 1354 billion by 2027 (Global Meat Industry Value Projection, 2021–2027|Statista, 2022). Thus, the market share of PBMAs is, and is estimated to remain, significantly lower than that of the meat market.

**Figure 2:**
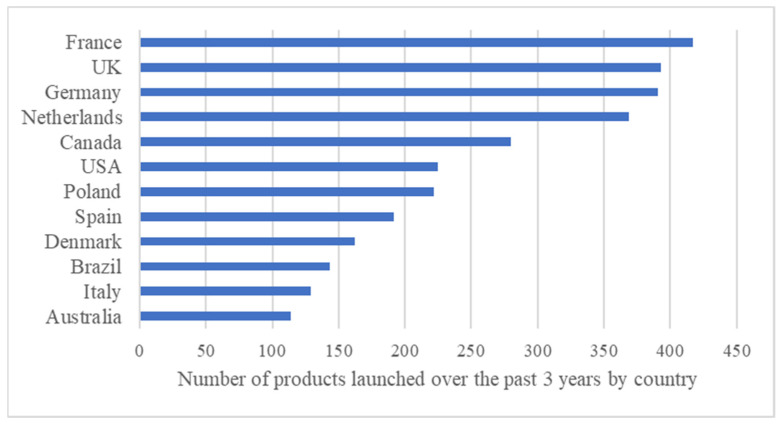
Twelve most active countries in PBMA launches over the past three years (2019–2021). Note: Each bar represents the total number of PBMAs’ launches between January 2019 and December 2021. Abbreviations: PBMAs, plant-based meat alternatives.

Considering the 2019–2021 period, new PBMA products were mostly launched in France, with 417 new launches (8.4%), followed by the UK (*n* = 393; 7.9%) and Germany (*n* = 391; 7.9%). The top twelve most active markets in this sector are represented in Figure 2. This figure underlines that European and northern American countries, along with Brazil and Australia, have been more active in launching plant-based meat alternatives during the past few years, showing an increasing interest in meat substitutes in these countries.

**Figure 3:**
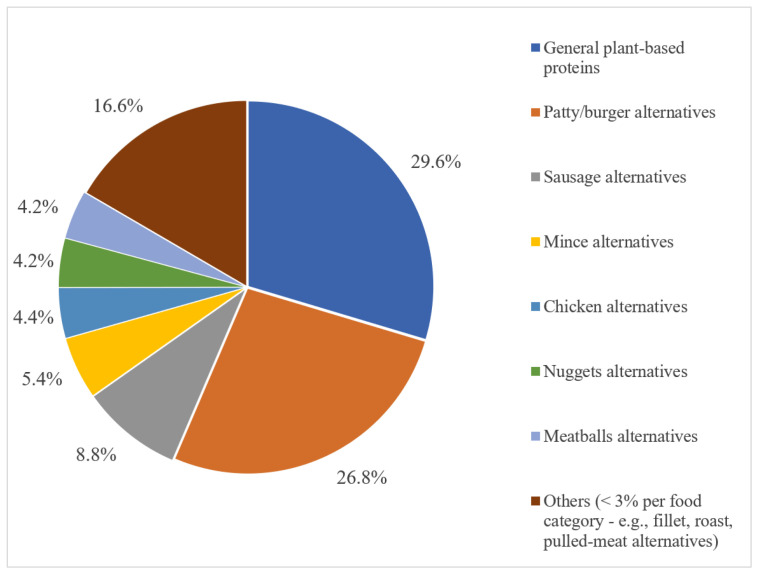
Food category distribution of PBMAs launched over the past three years (2019–2021). Abbreviations: PBMAs, plant-based meat alternatives.

In the global market, the most represented food categories were general plant-based proteins (*n* = 1469; 29.6%)—meaning foods that do not intend to mimic an existing meat product (e.g., burgers, sausages, nuggets, or meatballs) but can still be considered meat substitutes, as they are protein-rich plant foods (e.g., “teriyaki tofu” and “fried gluten with peanuts”)—and patty/burger alternatives (*n* = 1331; 26.8%). Every other food category alone—such as sausage, mince, or nugget alternatives—does not represent more than 9% of the total launches, as illustrated in Figure 3.

In terms of the highest sales value in EUR and the growth rate, according to a recent study of the Smart Protein project [76] using Nielsen Retail Scanning Data, the UK and Germany lead the sector of PBMAs, i.e., sausages, burger patties, and cold cuts. However, differences in the categories exist between countries; for example, plant-based sausages lead the market segment in the UK, whereas, in Germany, the top category is plant-based refrigerated meat (burger patties, nuggets, minced, etc.), followed by plant-based cold cuts and meat spreads and plant-based sausages.

Regarding the ingredients, we used the data from Mintel to identify which foods are most widely used as the first ingredient. When *water* was reported to be the first element in the list (*n* = 1605; 32.3%), we considered the second one. Using this strategy, we identified 1914 products (38.6%) containing *soy-based components*—e.g., soybean curd, proteins, or flour—as the first ingredient. After *soy*, *wheat* (*n* = 520; 10.5%) and other *pulses* (*n* = 702; 14.1%), such as kidney beans, black beans, peas, chickpeas, and lentils, were predominantly used as the first ingredient, followed by *mushrooms* (*n* = 134; 2.7%) and *jackfruit* (*n* = 86; 1.7%).

**Figure 4:**
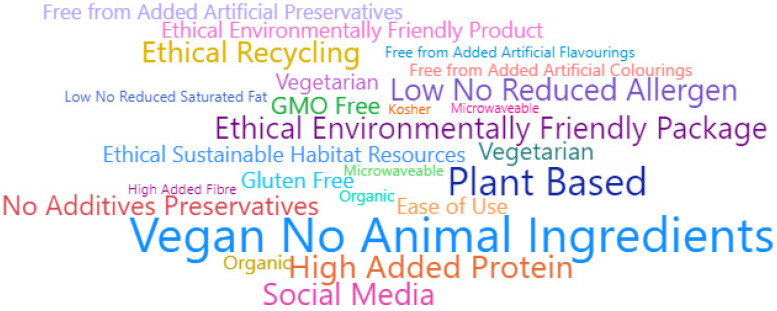
Word cloud of the top 20 claims employed in PBMA products launched over the past three years (2019–2021). Abbreviations: PBMA, plant-based meat alternative. Note: A word cloud is a visual representation of word frequency and value. The “Social Media” claim indicates the presence on the packaging of a logo/claim to entice consumers to join the company’s social media community and follow its channel/website.

In terms of information provided on the packaging, a total of 120 different claims were identified. Out of 4965 products, 2849 (57%) included the “Vegan/No Animal Ingredients” claim, and 2099 (42%) reported the “Plant Based” claim. In addition, in line with Cutroneo et al. [19], the most common nutrition claim was the “High/Added Protein” statement (*n* = 1616; 33%). A graphic presentation of the claims is represented in Figure 4.

Finally, Mintel’s Global New Product Database has been a practical tool to obtain a global overview of PBMA market trends. The data retrieved and analyzed from the database showed that plant-based meat alternatives can widely differ in terms of the food category, ingredients, and/or claims. However, despite these several variations, the increasing trend in product launches—especially in Western countries—highlights a promising global trend to support the transition toward a plant-based diet. However, as previously highlighted in this section, market share differences between the meat and PBMA sectors are still notable, and meat revenue forecasts do not foresee any declining trends. These data underline that the growing market of meat substitutes does not significantly affect the meat market. Therefore, PBMAs are still weak substitutes for animal-based products, as they are often complementary to meat rather than meat replacers [77]. Previous studies also showed that regular meat consumers are less likely to choose plant-based items over beef than people declaring that they follow different diets (e.g., vegan, flexitarian, or vegetarian) [78]. Thus, in order to support a dietary shift toward meat reduction, it is critical to study and test strategies that could steer meat eaters’ choices toward plant-based diets and support the growing market of PBMAs.

## 7. Discussion and Conclusions

Plant-based foods that replace animal foods, such as meat, but also dairy, and even fish and eggs, are gaining increased attention as possible substitutes that can facilitate the transition toward sustainable healthy diets. The idea of processing plant-based ingredients to obtain protein-based foods is not a new concept for consumers since many products, such as tempeh, tofu, and seitan, have been available on the market for hundreds of years [4], especially in Asian countries. However, these products were not intended to be meat substitutes per se and have never become mainstream in Western countries. A possible explanation could be that these products have mostly been targeted at vegetarians or vegans without any explicit reference to their animal counterparts.

Nevertheless, the development of the so-called “meat alternatives” sector is gaining more and more attention due to growing concerns over the environmental impacts of the food system [5] and the increasing awareness of the detrimental impacts of high meat consumption on human health [79].

In the last several years, hundreds of meat-like substitutes, such as plant-based burgers, have been developed and launched globally on the market to imitate the traditional beef burger using either 100% plant-based ingredients or a mix of both meat and plant-based ingredients, i.e., “hybrid meat products”. Although this latter category is not suitable for vegetarians and vegans, these hybrid meat alternatives could exploit consumer barriers to PBMAs (e.g., low sensory quality) and lead to the first approach to reducing meat consumption.

The growing demand for PBMAs has driven the development of ground-breaking process technologies and novel ingredients that can help to obtain products with meat-like sensory attributes that have the potential to attract non-vegetarian consumers [52]. However, many of these new meat alternatives are highly complex products in terms of ingredients/formulations and require technological investments [80]. For instance, one limitation of using plant proteins as meat substitutes is the challenge of preserving the shape while dealing with the high risk of crumbling [56]. For this reason, as of now, most of these proteins have been employed either as a meat ingredient substitute (e.g., in the shape of mince) or as parts of food products (pizza, sauces, etc.) and have not been consumed on their own [55]. Currently, a new line of familiar alternatives to traditional meat products or dishes, such as imitation-meat burgers, has been launched in supermarkets and restaurants [81].

While targeting young flexitarians and omnivores is seen as the key to ensuring growing sales of plant-based meat alternatives in the future [82,83], there is still the need to investigate whether and how the sensory appeal will be a barrier for the second generation of plant-based meat alternatives among these consumers [5].

To achieve acceptability among non-vegetarian consumers, plant-based foods should resemble the texture, flavor, appearance, aroma, and taste of authentic meat products. However, the long list of unfamiliar ingredients used to mimic meat sensory properties leads to different nutrition values of these products compared to animal meats. As a result, even if PBMAs are similar to meat in terms of sensory experience, they cannot be considered a nutritional replacement for animal products [4]. Thus, further studies are needed not only to monitor the nutritional quality of new plant-based meat products on the market but also to investigate the impact of this substitution on human health markers. In addition, adequate nutritional education programs to improve consumers’ knowledge and awareness about the differences between animal- and plant-based products are required [19].

Moreover, the discussion on whether manufacturers should describe PBMA products using references to their animal counterparts (e.g., “tastes like meat”), which could create positive expectations for meat consumers [5,62], is still under debate. Specifically, after the recent commercial success of several PBMAs, a strong debate has started on how to label/name such products. For example, in the EU, a regulation clarifying whether “meat-sounding” labels for PBMAs should be allowed does not exist yet. This outcome will probably impact consumer preferences, as shown in a recent study by Demartini et al. [84], in which consumers’ perceptions of tastiness and healthiness and their willingness to buy plant-based meatballs were negatively affected by the vegan labeling.

As we reported, the sector of PBMAs is launching products on the market that mimic their animal counterparts, and the term “meat substitutes” seems to imply that people will stop eating meat [4]; however, it is more likely that individuals will consume both traditional and non-traditional meat alternatives. In this scenario, PBMAs may be a useful tool to reduce animal products, especially for populations that consume too much animal meat according to dietary recommendations. We might also expect PBMAs to be regarded as an intermediate phase on our way to (semi-)plant-based diets, in which unprocessed plant-based foods and recipes would take center stage. Achieving this kind of diet would mean that our food habits have really gone beyond meat.

Finally, future studies should consider calls for collaboration, particularly among stakeholders of the food supply chain (i.e., industries and food services) and the scientific community (i.e., nutritionists and dietitians, food technologists, and consumers scientists), to facilitate the transition toward healthier and more sustainable plant-based protein sources.

## Data Availability

Data will be available upon request.

## Appendix A

**Table A1:** Search criteria considered in Mintel database.

Search Variable	Criteria
Category	“Food”
Sub-category	“Meat Substitutes” (with “Format Type” matching one or more of the following: ball; block/cubed; burger; sausage; fillet; shredded/minced; sliced; other)
Date Published	within the “last three complete years” (January 2019–December 2021)
Region/Market	“Across all regions/markets”
